# Association Between Food Security Status and Adherence to Mediterranean Diet Among Adults in Saudi Arabia: A Cross-Sectional Study

**DOI:** 10.3390/foods15101777

**Published:** 2026-05-18

**Authors:** Mahitab Hanbazaza, Maram Bajunayd

**Affiliations:** Department of Food and Nutrition, Faculty of Human Sciences and Design, King Abdulaziz University, Jeddah 22258, Saudi Arabia; mbajunayd@stu.kau.edu.sa

**Keywords:** mediterranean diet, food insecurity, adults, Saudi Arabia

## Abstract

Food insecurity has been associated with poorer diet quality; however, limited evidence exists on the association between food insecurity and adherence to the Mediterranean diet in Saudi Arabia. This cross-sectional study, conducted among 577 Saudi adults between February and June 2025, examined the association between food security status and adherence to the Mediterranean diet in this region. Data were collected through a self-administered questionnaire that included sociodemographic characteristics, the Food Insecurity Experience Scale, and the Mediterranean diet adherence score. Most participants were food secure (73.0%), and only 12.7% demonstrated high adherence to the Mediterranean diet. The food insecurity score was inversely related to Mediterranean diet adherence (B = −0.107, 95% CI −0.191 to −0.024; *p* = 0.012); however, the magnitude of the association was small. Age, marital status, and monthly income were also significantly associated with food security status (*p* < 0.005). Although most of the participants were food secure, overall adherence to the Mediterranean diet was low, with only a small proportion demonstrating high adherence. These findings suggest that socioeconomic factors, particularly income, may be associated with both food access and diet quality. Further research is needed to inform strategies aimed at improving access to affordable healthy foods and promoting healthier dietary patterns, especially among young adults and low-income individuals.

## 1. Introduction

The Mediterranean diet (MedDiet) is one of the most extensively studied dietary patterns, consistently linked to improved diet quality and favorable health outcomes in adults [1,2]. Evidence from observational studies and systematic reviews indicates an association between greater adherence to the MedDiet and a lower risk of cardiovascular disease, type 2 diabetes, mental health conditions, and all-cause mortality, as well as better health-related quality of life [2,3,4]. Although the MedDiet originates from Mediterranean countries, it has increasingly been recognized as a globally relevant dietary pattern for healthy and sustainable eating. However, despite these well-established benefits, adherence to the MedDiet remains suboptimal in many adult populations, suggesting that factors beyond individual dietary knowledge may influence its adoption [5]. Studies have indicated that adherence to the MedDiet is generally low, particularly in Arab countries [6]. In one study, across the Gulf countries (Saudi Arabia, Oman, Kuwait), 44% of individuals exhibited low adherence, whereas 22.4% demonstrated high adherence [7]. Specifically in Saudi Arabia, high adherence has been reported at just 10.1% [8]. In Mediterranean countries such as Italy and Spain, adherence has been reported to be 13.3% and 11.0%, respectively [9,10]. This reduction has been linked to economic and social changes, with several regions shifting toward less healthy dietary patterns [11].

Dietary behaviors are shaped by a complex interplay of social, economic, and environmental factors. Prior research has shown that socioeconomic position, food affordability, and access to nutritious foods substantially influence dietary quality and long-term adherence to healthy eating patterns [12]. In many settings, including Saudi Arabia, these factors are occurring alongside a broader nutrition transition characterized by increased consumption of energy-dense and ultra-processed foods and declining adherence to traditional dietary patterns. For many adults, declining purchasing power has limited access to nutrient-dense foods, including fresh fruits and vegetables, fish, and olive oil. As a result, reliance on lower-cost, energy-dense, and nutrient-poor foods may contribute to poorer overall diet quality [12].

In this context, food security is considered a determinant of health, influenced by economic, geographic, and social factors, and affecting health outcomes both directly and indirectly [13]. Food insecurity is defined as a lack of access to sufficient food necessary for growth and health [14]. Food and Agriculture Organization of the United Nations (FAO) data indicate that 28.0% of the global population was affected by moderate-to-severe food insecurity in 2023–2024. The prevalence of food insecurity varies by region; 24.8% in Asia, 8.7% in North America and Europe, and 40.2% in Arab countries [15,16]. Several studies have shown that food insecurity is associated with poorer diet quality and lower adherence to healthy dietary patterns, including the MedDiet [17]. Adults experiencing food insecurity are more likely to rely on energy-dense, nutrient-poor foods due to economic constraints, which may limit adherence to dietary patterns rich in fruits and vegetables, whole grains, and healthy fats [17,18].

However, much of the existing literature has examined dietary quality or food security separately, and evidence on their combined association in adult populations remains limited. Despite the well-established health benefits of the MedDiet, its adherence in non-Mediterranean countries remains understudied. In Saudi Arabia, rapid dietary changes associated with the nutrition transition have led to increased consumption of energy-dense and ultra-processed foods, alongside a decrease in diet quality. Examining adherence to the MedDiet in this context provides an opportunity to assess its applicability, identify potential barriers, and understand how cultural factors and food insecurity may influence dietary behaviors. Therefore, this study aimed to examine the association between food security status and adherence to the MedDiet among adults. Clarifying this association may help inform public health strategies aimed at improving diet quality and supporting vulnerable populations.

## 2. Materials and Methods

### 2.1. Study Design

This cross-sectional study examined the association between food security status and adherence to the MedDiet among adults in Saudi Arabia. The self-administered questionnaire was distributed nationwide across Saudi Arabia via Google Forms between February and June 2025. The questionnaire was also shared on social media platforms, including X, WhatsApp, Snapchat, and Telegram, as well as via university email.

To minimize duplicate responses, the survey restricts each device/IP address to one response. In addition, responses were screened for completeness and potential duplication before analysis. The first part of the questionnaire included a brief introduction explaining the study’s purpose, voluntary participation, and the right to refuse or withdraw at any time.

### 2.2. Study Population and Sample

The study sample comprised male and female adults aged 18–64 years in Saudi Arabia. Participants were recruited using convenience sampling. Individuals aged 65 years and above were excluded, as older adults represent a distinct population group with different nutritional needs, health conditions, and food access challenges. Also, individuals following medically prescribed diets were excluded. The total adult population aged 18–64 years in the Kingdom of Saudi Arabia is estimated at 21,229,785 [19]. According to a 95% confidence level and a 5% margin of error, the minimum required sample size for this study was approximately 385 participants [20]. However, this calculation was used as a general guideline for sample adequacy and does not indicate representativeness of the target population, as a non-probability convenience sampling approach was used. A total of 600 responses were received, and 23 were excluded due to missing data in the food security section.

This study was approved by the ethics committee of the Faculty of Medicine at King Abdulaziz University (Approval number: No. 48-25) and conducted in accordance with the ethical principles of the Declaration of Helsinki. Informed consent was obtained from all participants before data collection, with full consideration of their rights.

### 2.3. Questionnaire Development

The questionnaire was developed using previously validated instruments. Therefore, formal psychometric validation was not the primary aim of this study. However, a pilot test was conducted to assess face and content validity to ensure clarity, cultural appropriateness, and contextual relevance. Content validity was evaluated via review by three associate professors in the field of food and nutrition. These professors assessed the questionnaire items to ensure they aligned with, were relevant to, and were appropriate for the study’s objectives. To assess face validity, we administered the questionnaire to five individuals from the general population who shared characteristics with the target sample, evaluating the language’s clarity, readability, consistency, and comprehensibility, and estimating completion time. Responses from this pilot group were excluded from the final analysis.

### 2.4. Measures

#### 2.4.1. Sociodemographic Characteristics

Sociodemographic variables were assessed, including gender, age, geographic region, education level, marital status, and monthly income. Age was categorized into groups (18–24, 25–34, 35–44, 45–54, 55–64). Education level was classified as (secondary or less, diploma, bachelor’s degree, postgraduate). Marital status was categorized as (single, married, divorced, widowed). Monthly income was categorized into the following groups: <4000 SAR, 4000–<7000 SAR, 7000–<10,000 SAR, 10,000–< 13,000 SAR, and ≥13,000 SAR. The geographic region was classified according to the main regions of Saudi Arabia (Western, Central, Eastern, Northern, and Southern).

#### 2.4.2. Food Security Assessment

Food security status was assessed using the validated Food Insecurity Experiences Scale survey module (FIES-SM) [21]. The Arabic version was obtained from the FAO website and validated for use in Arabic-speaking populations [21,22]. Although it has not been specifically validated in the Saudi context, its use is supported by previous research conducted in similar cultural and linguistic settings [22]. The instrument comprises 8 items assessing participants’ experiences of food insecurity over the past month, with responses coded as yes/no. Scores range from 0 to 8, and participants were categorized into four levels: 0 = food security; 1–3 = mild food insecurity; 4–6 = moderate food insecurity; and 7–8 = severe food insecurity. For analysis purposes, food insecurity categories (mild, moderate, and severe) were combined into a single group, the “food insecure” category. This approach was used to enable statistical modeling and ensure an adequate sample size within each group.

#### 2.4.3. Mediterranean Diet Adherence

Adherence to the MedDiet was assessed using the Mediterranean Diet Adherence Scale (MEDAS) [23]. This validated scale has been adapted for use in many countries and has demonstrated strong construct validity [24]. The questionnaire originally included 14 items. One question on wine consumption was removed because of alcohol prohibitions in Islamic regions, reducing the scale to 13 items. An existing Arabic version of the MEDAS, which was previously used in an Arabic-speaking population, was adopted in this study [7]. Scores range from 0 to 13, with higher scores indicating greater adherence to the MedDiet. The participants’ scores were categorized as low (0–5), medium (6–7), or high (8–13), based on cut-offs adopted from a previous study that used similar modified versions of the MEDAS among adults in Saudi Arabia [7]. Internal consistency of the modified 13-item MEDAS was assessed using Cronbach’s alpha. In the present sample, internal consistency of the modified 13-item MEDAS was low, with Cronbach’s alpha = 0.389.

### 2.5. Statistical Analysis

Statistical analyses were performed using SPSS version 26.0. Descriptive statistics, including frequencies and percentages, were used to summarize the sociodemographic characteristics and key variables relevant to the study. These variables included sex, age, geographical region, educational level, marital status, and monthly income. Continuous variables, including MedDiet adherence and food insecurity scores, were presented as means, standard deviations, and ranges (Mean ± SD [range]).

The association between sociodemographic characteristics and food security status was analyzed using cross-tabulation and the chi-square and Fisher’s Exact tests for categorical variables. A generalized linear model was used to assess the association between food insecurity and MedDiet adherence. In this analysis, both variables were treated as continuous, with higher food insecurity scores indicating worse food security. Two models were used: Model 1 (crude) included only the food insecurity score; Model 2 was adjusted for sex, age group, region, educational level, marital status, and monthly income. The generalized linear model assumed a Gaussian distribution with an identity link function. Model assumptions, including linearity, homoscedasticity, and normality of residuals, were assessed using residual plots and Q–Q plots. Multicollinearity was evaluated using variance inflation factors (VIF). Missing data were handled using complete-case analysis. A *p*-value < 0.05 was considered significant.

## 3. Results

### 3.1. Sociodemographic Characteristics of Study Participants

After excluding participants with missing data in the food security section, 577 adults were included in this study. Of this sample, 55.8% were female. The largest group (29.1%) was aged 25–34 years. The substantial majority (81.6%) lived in the Western region. Furthermore, the most common education level was a bachelor’s degree (52.5%), and 53.9% were married. In addition, 38.1% reported a monthly income of less than 4000 SAR (1065.83 USD). Regarding adherence to the MedDiet, most respondents (51.6%) reported low adherence, and only 12.7% reported high adherence. Regarding food security status, most individuals (73.0%) were food secure, whereas 27% were food insecure, with 14.4% having mild food insecurity, 10.4% moderate, and 2.2% severe. See Table 1 and Table 2.

### 3.2. Bivariate Association Between Sociodemographic Characteristics and Food Security Status

**Figure 1:**
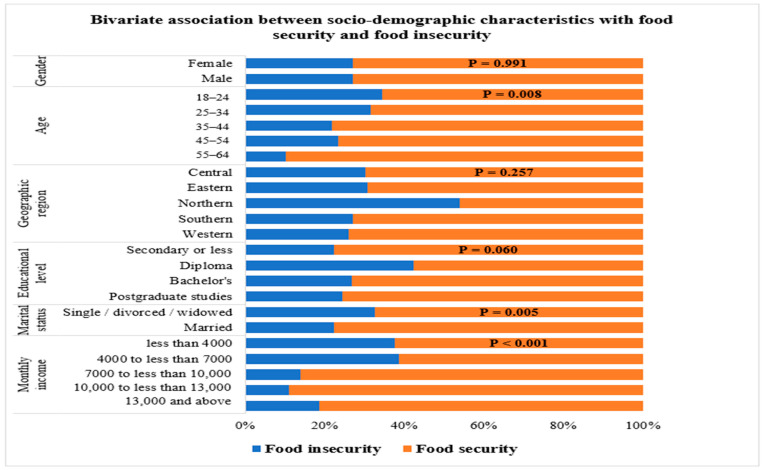
Bivariate association between socio-demographic characteristics and food secure status.

Table 3 and Figure 1 present the association between sociodemographic characteristics and food security status among the study participants. Age, marital status, and monthly income were significantly associated with food security status. Regarding age, participants aged 18–24 and 25–34 years had the highest prevalence of food insecurity (34.3% and 31.5%), compared with 10.3% among adults aged 55–64 (*p* = 0.008). A significant association was also observed between marital status and food security (*p* = 0.005). Single, divorced, or widowed participants were more likely to experience food insecurity (32.7%) than married participants (22.2%). Monthly income showed the strongest association with food security (*p* < 0.001). Participants earning less than 4000 SAR per month reported the highest prevalence of food insecurity (37.7%). In comparison, those earning 10,000 SAR or more exhibited much lower rates (11.1–18.7%). In contrast, sex, geographic region, and educational level were not associated with food security status.

### 3.3. Bivariate Association Between MedDiet Adherence and Food Security Status

**Figure 2:**
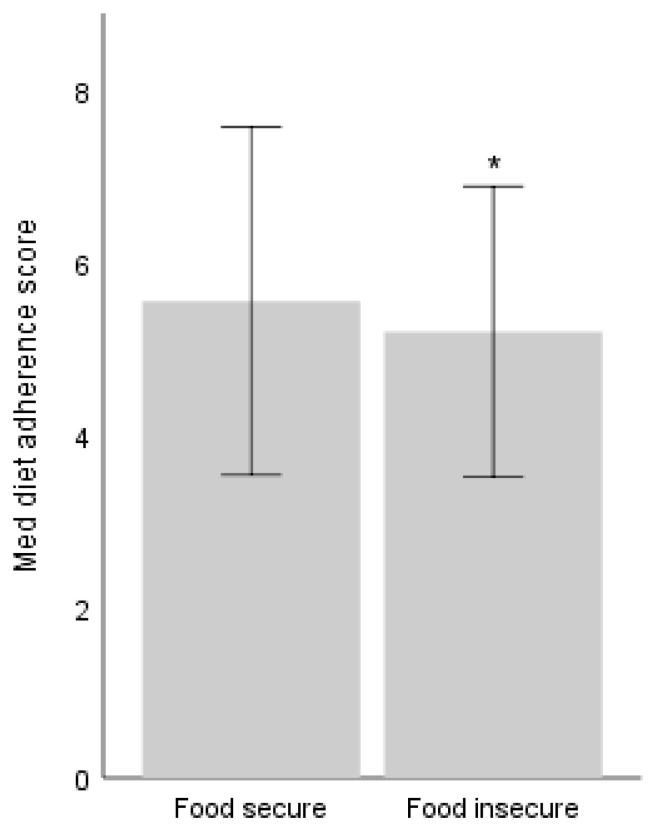
Difference in Mediterranean diet adherence score between food-secure (n = 421) and food-insecure people (n = 156). * denotes statistically significant difference (*p* < 0.05).

As shown in Table 4 and Figure 2, adherence to the MedDiet was slightly lower among food-insecure participants than among food-secure participants (5.19 ± 1.69 vs. 5.54 ± 2.02; *p* = 0.034). However, the total difference was small, 0.35 points on a 13-point scale. The approximate Cohen’s d was 0.18, indicating a small effect size. The distribution of adherence levels (low, medium, and high) did not differ significantly between the two groups (*p* = 0.089).

### 3.4. Association Between Food Insecurity Score and MedDiet Adherence Score

Because several categorical covariates (sex, age group, region, educational level, marital status, and monthly income) were included, a generalized linear model was used to assess the association between food insecurity and MedDiet adherence. Both variables were continuous, with higher values indicating worse food security. Two models were used: Model 1 (crude) included only the food insecurity score; Model 2 was adjusted for sex, age group, region, educational level, marital status, and monthly income. In the crude model, the food insecurity score was inversely associated with the MedDiet adherence score. Each one-point increase in food insecurity score was associated with a 0.107-point lower MEDAS score, B = −0.107, 95% CI −0.191 to −0.024; *p* = 0.012. The crude model explained 1.1% of the variance in MEDAS score, R^2^ = 0.011.

In the adjusted general linear model, food insecurity score remained significantly associated with MEDAS score after adjustment for gender, age group, geographical region, educational level, marital status, and monthly income. Each one-point increase in food insecurity score was associated with a 0.101-point lower MEDAS score, B = −0.101, 95% CI: −0.189 to −0.014, *p* = 0.023. The full adjusted model was statistically significant, F (18, 558) = 2.128, *p* = 0.004, but explained only a small proportion of the variance in MEDAS score, R^2^ = 0.064; adjusted R^2^ = 0.034. The effect size for food insecurity was small, partial η^2^ = 0.009. As shown in Table 5.

## 4. Discussion

This study examined the association between food security status and MedDiet adherence among adults in Saudi Arabia. Overall, although most participants were food-secure, only a small proportion showed high adherence to the MedDiet. Adherence to the MedDiet was generally low, and significantly lower among food-insecure participants than among food-secure individuals. A significant inverse association was observed between food insecurity and adherence to the MedDiet; however, the magnitude of the association was small, suggesting that individuals experiencing food insecurity may face greater difficulty maintaining a healthier dietary pattern. In addition, several sociodemographic factors, including age, marital status, and monthly income, were significantly associated with food security status. These results highlight the important role of socioeconomic factors in shaping both food access and dietary behaviors within the Saudi population.

In the present study, adherence to the MedDiet was generally low to moderate, with only a small proportion of participants reporting high adherence. These results are consistent with previous studies conducted in Saudi Arabia, which have also demonstrated low adherence to the MedDiet [8,25]. A study comparing adherence to the MedDiet across Mediterranean and non-Mediterranean populations found that people in non-Mediterranean countries reported lower adherence [26]. In an Italian study, 13.3% of participants demonstrated high adherence to the MedDiet [9], whereas in a study conducted in Spain, only 11% adhered to this dietary pattern [10]. Previous research has also suggested that higher education may be associated with greater adherence to healthy dietary patterns, including the MedDiet [25]. A study conducted in Saudi Arabia found that only 10.1% of participants adhered to the MedDiet [8]. The relatively low adherence observed in the current study may reflect limited awareness of the MedDiet in the Saudi population [8]. While the MedDiet provides a valuable framework for healthy eating, its use in the Saudi context may be influenced by cultural dietary practices and food preferences.

Our findings reveal a significant inverse association between food insecurity and MedDiet adherence; however, the magnitude of the association was small, suggesting that additional environmental, cultural, and behavioral factors may also influence dietary patterns. This is consistent with findings from a previous review conducted among adults in Mediterranean countries, which indicated that food insecurity is associated with lower diet quality and reduced intake of nutrient-dense foods, including vegetables, fruit, and whole grains [27]. Individuals experiencing food insecurity may face financial constraints that limit their ability to purchase healthier foods, particularly those that are relatively more expensive, such as fresh produce, fish, and olive oil, which are key components of the Mediterranean dietary pattern. Thus, food-insecure households may rely more heavily on inexpensive, energy-dense foods that provide greater caloric value at lower cost but are less consistent with recommended dietary patterns [17]. Similar findings have been reported in other countries, including Portugal, Turkey, and Spain, where higher levels of food insecurity were associated with lower adherence to the MedDiet [17,28,29].

These findings suggest that economic constraints and food affordability play an important role in shaping dietary behaviors. Previous research has also shown that adherence to the MedDiet may be associated with higher food costs, making low-income individuals more vulnerable to deviating from this dietary pattern [30].

However, the distribution of adherence levels (low, medium, and high) did not differ significantly between the groups. This finding may reflect the generally low adherence to the MedDiet within the study population. In Saudi Arabia, adherence to the MedDiet is generally lower than in Mediterranean countries [26,31]. Studies have shown that only a small percentage of the Saudi population adheres to healthy dietary guidelines. Adults tend to consume high quantities of processed foods, fats, sugars, and high-calorie foods, and lower intakes of vegetables, fruits, and seafood; this dietary pattern is linked to lower adherence to the MedDiet [32,33,34] and is culturally ingrained and widely followed. Reduction in adherence to the MedDiet, regardless of food security status, may be influenced by cultural eating habits, food availability, and the ongoing nutrition transition, characterized by increased consumption of processed and convenience foods [35]. This reflects a broader dietary shift toward more Westernized dietary patterns, characterized by higher intake of animal-based foods and lower consumption of plant-based foods. The observed associations may also reflect broader food environment factors, including the availability, accessibility, and marketing of foods, which can shape dietary choices independently of individual preferences.

The findings also revealed that several sociodemographic factors were associated with food security status. Younger adults were found to be more likely to face food insecurity than older participants, consistent with the findings of a previous study in which younger adults (<22 years) were found to be more likely to experience food insecurity than older adults, often due to financial instability and transitional life stages, such as higher education or early career development [36]. Although food affordability was not directly measured in the current study, these findings may reflect the influence of broader socioeconomic and food environment factors that shape food access and dietary behaviors.

Marital status was also significantly associated with food security, with single, divorced, or widowed participants being more likely to report food insecurity than married individuals. Previous studies have shown that married people with higher incomes and education tend to be food secure, as education and income improve access to adequate food, and family stability helps reduce food insecurity [37,38]. In addition, marriage may provide both financial stability and social support through shared household resources, potentially reducing vulnerability to food insecurity. Family life, shared meals, and various cooking methods promote healthier habits, including adherence to the MedDiet [39,40]. Monthly income was strongly associated with food security, with a high rate of food insecurity among low-income participants. This finding supports previous research showing that limited financial resources are a key factor in food insecurity [41,42]. Low-income households often face greater challenges in accessing sufficient nutritious food due to higher food costs relative to their income [43,44].

## 5. Strengths and Limitations

This study contributes to the limited literature by examining the association between food security and adherence to the MedDiet in the Saudi population.

However, several limitations should be considered. First, the study relied on a self-report questionnaire to assess adherence to the MedDiet, which may be subject to self-report and social desirability bias. Second, because of the cross-sectional design, causal relationships cannot be established. Third, the use of convenience sampling and online recruitment may have introduced selection bias, as participants were more likely to be younger, more educated, and have regular internet access. In addition, most participants were from the Western region of Saudi Arabia, which may limit the generalizability of the findings to other regions. Furthermore, the study did not evaluate food prices, food availability, or food environment factors, which may influence adherence to the MedDiet. Although the MEDAS was based on previously validated tools, the adapted 13-item version used in this study has not been validated in the Saudi context, which may affect measurement accuracy and limit the available psychometric evidence among Saudi adults. The internal consistency of the adapted 13-item MEDAS was low in the current sample (Cronbach’s α = 0.389), which may reflect limited intercorrelation among items. As the MEDAS assesses multiple dietary behaviors rather than a single construct, lower internal consistency may be expected and does not indicate poor validity. However, this low reliability may also indicate potential measurement error, which could weaken the observed associations and limit comparability with studies using the original validated MEDAS instrument. Additionally, the study did not account for several potentially important behavioral and health-related confounders, such as physical activity, health status, and other lifestyle factors, which may influence dietary patterns and food insecurity. Finally, categorizing food insecurity as a binary variable may have reduced the ability to detect differences across severity levels. Therefore, the findings should be interpreted with caution.

## 6. Implications

The findings of this study highlight an inverse association between food security and adherence to the MedDiet in Saudi Arabia. Public health strategies that aim to improve access to nutritious foods and promote healthy dietary behaviors may help address differences in diet quality among vulnerable groups. Supporting long-term adherence to the MedDiet and encouraging lifestyle changes may contribute to reducing processed food intake, increasing the consumption of fresh and local foods, and supporting sustainability. These findings may inform the development of targeted interventions aimed at enhancing healthy dietary patterns and overall health and quality of life among vulnerable groups. Future strategies could include educational campaigns and workplace programs to encourage adherence to the MedDiet as part of a sustainable eating pattern.

## 7. Conclusions

This study evaluated the prevalence of food insecurity and adherence to the MedDiet among adults in Saudi Arabia and examined the association between these two variables. Although most participants were food-secure, overall adherence to the MedDiet was low, with only a small proportion showing high adherence. A significant inverse association was found between food insecurity and adherence to the MedDiet; however, the magnitude of the association was small, suggesting that factors beyond food security may also influence dietary patterns. Additionally, age, marital status, and monthly income were significant sociodemographic factors associated with food insecurity.

These findings highlight potential socioeconomic constraints that may influence both food access and diet quality. Efforts to improve food security and promote affordable, healthy food choices may support healthier dietary behaviors among the Saudi population. Interventions that promote healthy habits and provide financial support are needed to make dietary diversity and healthier food choices financially accessible, especially for low-income and younger individuals. Further research using longitudinal and more representative study designs is needed to better understand the complex relationship between food insecurity and dietary patterns in Saudi Arabia.

## Figures and Tables

**Table 1:** Socio-demographic characteristics of study participants, *n* = 577.

Socio-Demographic Characteristics	Frequency (%)
**Gender**	
** Female**	322 (55.8%)
** Male**	255 (44.2%)
**Age**	
** 18–24**	137 (23.7%)
** 25–34**	168 (29.1%)
** 35–44**	147 (25.5%)
** 45–54**	86 (14.9%)
** 55–64**	39 (6.8%)
**Geographic region**	
** Central region**	43 (7.5%)
** Eastern region**	13 (2.3%)
** Northern region**	13 (2.3%)
** Southern region**	37 (6.4%)
** Western region**	471 (81.6%)
**Educational level**	
** Secondary or less**	63 (10.9%)
** Diploma**	52 (9.0%)
** Bachelor’s**	303 (52.5%)
** Postgraduate studies (Master’s and PhD)**	159 (27.6%)
**Marital status**	
** Divorced**	25 (4.3%)
** Married**	311 (53.9%)
** Single**	235 (40.7%)
** Widowed**	6 (1.0%)
**Monthly income**	
** Less than 4000**	220 (38.1%)
** 4000 to less than 7000**	70 (12.1%)
** 7000 to less than 10** **,** **000**	58 (10.1%)
** 10** **,** **000 to less than 13** **,** **000**	63 (10.9%)
** 13** **,** **000 and above**	166 (28.8%)

**Table 2:** Other characteristics of study participants, n = 577.

	Frequency (%) or Mean ± SD [Range]
**Adherence to the Mediterranean diet**	
**Adherence score**	5.45 ± 1.94 [0–13]
**Adherence classification**	
** Low adherence**	298 (51.6%)
** Medium adherence**	206 (35.7%)
** High adherence**	73 (12.7%)
**Food security**	
**Food security score ^1^**	0.95 ± 1.89 [0–8]
**Food security classification**	
** Food secure**	421 (73.0%)
** Mildly food insecure**	83 (14.4%)
** Moderately food insecure**	60 (10.4%)
** Severely food insecure**	13 (2.2%)

Note: ^1^ higher score corresponds to greater food insecurity, and a lower score corresponds to food security. Adherence scores range from 0 to 13, with higher scores indicating greater adherence to the MedDiet. The modified 13-item MEDAS showed low internal consistency in the present sample, with Cronbach’s alpha = 0.389.

**Table 3:** Bivariate association between socio-demographic characteristics and food security status.

Socio-Demographic Characteristics	Food Insecure (*n* = 156)	Food Secure (*n* = 421)	*p*-Value
**Gender**			0.991 ^a^
**Female**	87 (27.0%)	235 (73.0%)	
**Male**	69 (27.1%)	186 (72.9%)	
**Age**			0.008 ^a^
**18–24**	47 (34.3%)	90 (65.7%)	
**25–34**	53 (31.5%)	115 (68.5%	
**35–44**	32 (21.8%)	115 (78.2%)	
**45–54**	20 (23.3%)	66 (76.7%)	
**55–64**	4 (10.3%)	35 (89.7%)	
**Geographic region**			0.257 ^b^
**Central region**	13 (30.2%)	30 (69.8%)	
**Eastern region**	4 (30.8%)	9 (69.2%)	
**Northern region**	7 (53.8%)	6 (42.6%)	
**Southern region**	10 (27.0%)	27 (73.0%)	
**Western region**	122 (25.9%)	349 (74.1%)	
**Educational level**			0.060 ^a^
**Secondary or less**	14 (22.2%)	49 (77.8%)	
**Diploma**	22 (42.3%)	30 (57.7%)	
**Bachelor’s**	81 (26.7%)	222 (73.3%)	
**Postgraduate studies ^1^**	39 (24.5%)	120 (75.5%)	
**Marital status**			0.005 ^a^
**Single/divorced/widowed**	87 (32.7%)	179 (67.3%)	
**Married**	69 (22.2%)	242 (77.8%)	
**Monthly income**			<0.001 ^a^
**Less than 4000**	83 (37.7%)	137 (62.3%)	
**4000 to less than 7000**	27 (38.6%)	43 (61.4%)	
**7000 to less than 10** **,** **000**	8 (13.8%)	50 (86.2%)	
**10** **,** **000 to less than 13** **,** **000**	7 (11.1%)	56 (88.9%)	
**13** **,** **000 and above**	31 (18.7%)	135 (81.3%)	

Note: ^1^ Postgraduate studies education level includes master’s and PhD; ^a^. chi-square test, ^b^. Fisher’s Exact test. The three levels of food insecurity have been merged into a single classification.

**Table 4:** Differences in adherence to the Mediterranean diet between food-secure and food-insecure individuals.

Adherence to the Mediterranean Diet	Food Secure (*n* = 421)	Food Insecure (*n* = 156)	*p*-Value
**Adherence score ^a^** **, mean ± SD**	5.54 ± 2.02	5.19 ± 1.69 *	0.034
**Adherence classification ^b^** **, n (n%)**			
**Low adherence**	208 (49.4%)	90 (57.7%)	0.089
**Medium adherence**	153 (36.3%)	53 (34%)	
**High adherence**	60 (14.3%)	13 (8.3%)	

^a^ Difference between food secure and insecure was analyzed using an independent *t*-test. ^b^ Difference between food secure and insecure was analyzed using the Chi-square test. * denotes statistically significant difference (*p* < 0.05).

**Table 5:** Association between food insecurity score and Mediterranean diet adherence score (N = 577).

Model	B	95% CI	*p*-Value	R^2^	Adjusted R^2^	Effect Size
Model 1 (crude)	−0.107 *	−0.191, −0.024	0.012	0.011	0.009	small
Model 2 (adjusted)	−0.101 *	−0.189, −0.014	0.023	0.064	0.034	partial η^2^ = 0.009

Model 1 included food insecurity score only. Model 2 adjusted for gender, age group, geographical region, educational level, marital status, and monthly income. In Model 2, categorical covariates were entered as fixed factors, and food insecurity score was entered as a continuous covariate. Higher food insecurity scores indicate worse food security. MEDAS score was the dependent variable * denotes statistically significant association (*p* < 0.05).

## Data Availability

The original contributions presented in this study are included in the article. Further inquiries can be directed to the corresponding author.

## Abbreviation

The following abbreviation are used in this manuscript:

MedDiet	Mediterranean diet
